# Role of FoxO Proteins in Cellular Response to Antitumor Agents

**DOI:** 10.3390/cancers11010090

**Published:** 2019-01-14

**Authors:** Giovanni Luca Beretta, Cristina Corno, Nadia Zaffaroni, Paola Perego

**Affiliations:** Molecular Pharmacology Unit, Department of Applied Research and Technological Development, Fondazione IRCCS Istituto Nazionale dei Tumori, 20133 Milan, Italy; giovanni.beretta@istitutotumori.mi.it (G.L.B.); cristina.corno@istitutotumori.mi.it (C.C.); nadia.zaffaroni@istitutotumori.mi.it (N.Z.)

**Keywords:** FoxO, tumor, drug resistance

## Abstract

FoxO proteins (FoxOs) are transcription factors with a common DNA binding domain that confers selectivity for DNA interaction. In human cells, four proteins (FoxO1, FoxO3, FoxO4 and FoxO6), with redundant activity, exhibit mainly a positive effect on genes involved in cell cycle, apoptosis regulation and drug resistance. Thus, FoxOs can affect cell response to antitumor agent treatment. Their transcriptional activity depends on post-translational modifications, including phosphorylation, acetylation, and mono/poly-ubiquitination. Additionally, alterations in microRNA network impact on FoxO transcripts and in turn on FoxO levels. Reduced expression of FoxO1 has been associated with resistance to conventional agents (e.g., cisplatin) and with reduced efficacy of drug combinations in ovarian carcinoma cells. FoxO3 has been shown as a mediator of cisplatin toxicity in colorectal cancer. A requirement for FoxO3-induced apoptosis has been reported in cells exposed to targeted agents (e.g., gefitinib). Recently, the possibility to interfere with FoxO1 localization has been proposed as a valuable approach to improve cell sensitivity to cisplatin, because nuclear retention of FoxO1 may favor the induction of pro-apoptotic genes. This review focuses on the role of FoxOs in drug treatment response in tumor cells and discusses the impact of the expression of these transcription factors on drug resistance/sensitivity.

## 1. Introduction

Response of tumor cells to antitumor agents involves multiple pathways, and the cell fate depends on the balance between cell death-inducing and cell survival signals. A variety of sensors and effectors cooperate in determining treatment outcome, with the joint action of several pathways. One of the most relevant routes implicated in maintaining tumor cell survival is the Phosphatidylinositol-3-Kinase (PI3K)/Akt pathway, which acts downstream of the activation of growth factors, and can be hyperactivated through different mechanisms [1]. In this scenario, Forkhead box O (FoxOs) proteins, a family of transcription factors, can act as sensors and effectors of treatment, given their participation in the regulation of cell cycle progression, apoptosis, oxidative stress resistance, and DNA damage repair [2,3,4]. The genes induced in such processes include—among others— Cyclin Dependent Kinase Inhibitor 1B (KIP1, p27), Growth Arrest And DNA Damage Inducible (GADD45), Bcl-2-like protein 11 (BIM), Fas ligand (FasL), catalase, Manganese-dependent superoxide dismutase (MnSOD) and DNA Damage Binding protein 1 (DDB1). FoxOs represent key downstream signaling nodes in the PI3K/Akt pathway, which in turn negatively regulate the activity of FoxOs [5]. The major positive regulator of FoxOs is c-Jun N-terminal kinase (JNK), which can phosphorylate such transcription factors at sites different from those phosphorylated by Akt, upon cellular stress [6]. Additionally, FoxOs have been implicated in glucose metabolism and energy homeostasis [2,3]. Mammalian FoxOs include FoxO1, FoxO3, FoxO4 and FoxO6; the latter is primarily expressed in the central nervous system. The other FoxOs exert redundant activities in tissues, due to co-expression [7].

Non-coding RNAs are key regulators of gene expression and play multiple biological functions [8]. Specifically, microRNAs (miRNAs)—small non-coding RNAs able to negatively regulate gene expression—have been shown to participate in the regulation of the levels of FoxOs [9,10,11]. Since each miRNA can simultaneously target multiple factors, the concomitant regulation of additional genes beside FoxOs may influence the outcome of drug treatment.

Here, we review the recent studies regarding the involvement of FoxOs in cell response in terms of sensitivity and resistance to conventional antitumor agents, including DNA damaging agents, particularly cisplatin, and targeted agents, with specific reference to Receptor Tyrosine Kinases (RTK) inhibitors. The poorly understood role of FoxO1 in the efficacy of drug combinations is emphasized, because it may suggest effective therapeutic strategies for drug-resistant tumors.

## 2. Forkhead Transcription Factors: Overview and Role in Cancer

Originally identified in *Drosophila*, in which the mutated form resulted in a head fork-like conformation, FOX genes code for transcription factors referred as “winged helix” proteins, based on X-ray studies that revealed a DNA-binding domain composed of three α-helices and two loops resembling butterfly wings [12,13]. From the discovery of the founding gene, many genes of this family were identified, especially in vertebrates [14]. In humans, the family consists of more than 100 members (from FOXA to FOXS) classified based on sequence homology [14]. The family is characterized by a conserved DNA-binding domain (DBD), namely the forkhead box. Among the numerous proteins belonging to this family, the FoxO subgroup is implicated in regulating cellular proliferation, stress tolerance, metabolism and lifespan [13,15]. The DBD contains a common unique five-amino acid signature (i.e., GDSNS), conferring selectivity for DNA binding at (G/C) (T/A) AA (C/T) AA consensus sequences termed FOXO-responsive elements [15]. The five-amino acid signature grants all FoxOs a similar DNA binding specificity. FoxOs play several biological functions, spanning from the regulation of genes involved in cell proliferation and differentiation, to embryonic development and longevity [16].

Post-translational modifications, including phosphorylation, acetylation, and ubiquitination, control the activity/function of FoxO transcription factors, which in turn regulate FoxO target gene expression levels [15]. FoxO transcription factors are inhibited by Akt-mediated phosphorylation in response to insulin or growth factor stimulation, and the phosphorylation results in FoxO export from the nucleus into the cytoplasm with inhibition of FoxO-dependent transcriptional activity [17]. This feature puts FoxO proteins at the interface of crucial insulin and other growth factor signaling [18]. Akt-mediated FoxO1, FoxO3, and FoxO4 phosphorylation in three different sites favors the interaction with the 14-3-3 protein, which is responsible for the nuclear export of the transcription factors, affecting cell growth and survival [2,19,20]. Conversely, the Akt-mediated phosphorylation of FoxO6 at two sites inactivates the transcription factor and prevents its export from the nucleus [21]. Other factors are negative regulators of FoxOs, including the IκB kinase (IKK), the Dual-specificity Tyrosine-phosphorylation-regulated kinase 1A (Dyrk1A), the Serum and glucocorticoid-regulated kinase (Sgk), Casein kinase 1 (Ck1), and Extracellular signal-regulated kinase (Erk1/2) [2,22,23]. Besides, nutrient deprivation activates the AMP-activated protein kinase (AMPK), which activates FoxOs and the expression of genes implicated in stress resistance and energy metabolism [23]. Oxidative-stress activates JNK or the Mammalian Ste20-like kinase (Mst1) that trigger FoxO phosphorylation and translocation into the nucleus [6].

Besides phosphorylation, FoxOs undergo additional post-translational modifications such as acetylation or ubiquitination. Acetylation impacts on DNA binding and transcriptional activity. Oxidative-stress-mediated FoxO deacetylation (via the NAD-dependent deacetylase sirtuin-1, Sirt-1) or monoubiquitination increases FoxO DNA binding capability and transcriptional activity [24]. Conversely, the poly-ubiquitination of FoxO results in proteasome-mediated protein degradation [24].

FOXO expression is also tightly regulated by the action of miRNAs targeting FoxO mRNA transcripts. For instance, several miRNAs (e.g., miR-27, miR-9, miR-96, miR-182, miR-183, miR-370, miR-135b, miR-1269, and miR-411) control FoxO expression in various tumor types, including melanoma, Hodgkin’s lymphoma, osteosarcoma, hepatocellular carcinoma, prostate, bladder breast, endometrium and lung cancers, with an impact on cell proliferation and viability, migration and survival, as well as cell death [11]. In osteosarcoma cells, miR-135b induces proliferation and invasion by targeting FoxO1 [25]. Along this way, the miR-182 dependent reduction of FoxO1 levels renders prostate cancer cells more prone to proliferation and invasion [26]. The reduced expression of FOXO4 in colorectal and gastric cancers correlated with increased miR-499-5p and miR-127a levels [27]. Additionally, the decreased expression of FOXO4 inversely correlated with miR-150 level in cervical carcinoma [28,29]. Of note, by altering the levels of FoxO1, FoxO3, and FoxO4, different miRNAs have been reported to exert opposite functions on epithelial–mesenchymal transition (EMT). Among these, miR-130b, miR-181b-3p and miR-331-3p [30,31,32] favor EMT, whereas miR-622 and miR-34b/c repress invasiveness by suppressing EMT [33].

**Figure 1:**
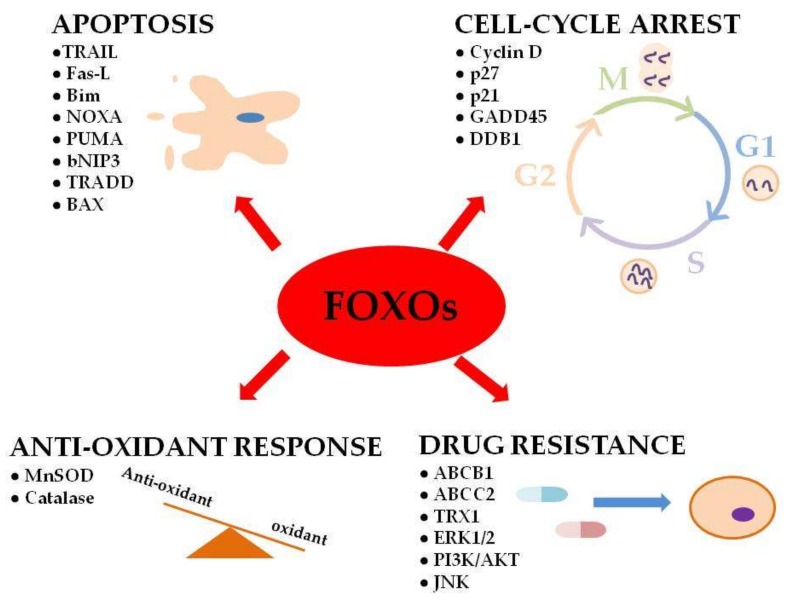
Implication of FoxO (FoxO proteins) functions in physiological processes and drug resistance in cancer.

In humans, FOXO genes were found to regulate important cellular functions (e.g., apoptosis, cell-cycle arrest and DNA repair) that are deregulated in cancer cells (Figure 1) [2,34].

The altered activity of transcription factors is critical in many human cancers [35,36,37,38] and several lines of evidence demonstrate that altered FoxO localization or function is associated with tumorigenesis and cancer progression [39,40,41]. The expression of FOXO in certain tumor cells arrests their growth in vitro and various tumor suppressors (e.g., Smad and p53) are FoxO partners influenced by its expression, activity and cellular localization [2,42]. This scenario suggests that FoxOs have tumor suppressor functions and that signaling pathways deregulated in tumors as well as miRNAs implicated in FOXO expression and regulation contribute to tumor development and progression [11]. As far as the signaling pathways are concerned, three main kinases, Akt, Erk1/2 and Ikk, mediate FoxO phosphorylation, inactivation and degradation. The PI3K/Akt pathway has been reported to control FoxOs repression in breast, cervical, lung, and thyroid cancers as well as in Hodgkin’s lymphoma [11]. Along this way, a deregulation of the canonical Mitogen-Activated Protein Kinase (MAPK)/Erk1/2 pathway was identified in breast cancer and in Hodgkin’s lymphoma, whereas the Ikk pathway emerged in acute myeloid leukemia and lung cancer as an important regulator of FoxO3 [11].

Given the biological functions of FoxOs, a role in response to conventional and target-specific antitumor agents is not unexpected.

## 3. FoxOs and Drug Response

Because the FOXO family of transcription factors regulates several transcriptional targets involved in processes that may affect tumor cell response to drug treatment, such as proliferation and apoptosis [7], FoxO proteins contribute to the regulation of tumor cell sensitivity and resistance. Indeed, the FOXO transcription factors participate in cellular response to conventional antitumor agents and therefore in drug resistance or sensitivity mainly by virtue of their activity as transcription factors whose function is regulated by protein abundance, post-translational modification and cellular localization.

FoxOs are known to mainly induce an increase in gene expression upon binding to DNA, although a repressive activity has also been evidenced [15]. For instance, transcriptional repression of D-type cyclins has been shown in relation to FOXO1A-mediated inhibition of cell cycle progression and transformation [43].

FOXO-activated genes comprise key players of cell cycle regulation (i.e., CDKN1A gene encoding p21^WAF1^), drug resistance (i.e., ABCB1), insulin signaling (i.e., 4E binding protein 1, 4EBP1) and apoptosis [7]. The expression of multiple pro-apoptotic factors including BH3-only proteins (BOPs; i.e., Bim, NOXA, PUMA, and bNIP3) and other cell death promoting genes (i.e., BAX, FasL, tumor necrosis factor-related apoptosis inducing ligand (TRAIL), and tumor necrosis factor receptor-associated death domain (TRADD)) is activated by FOXOs, which participate in promoting apoptosis in a mitochondria-independent and -dependent manner, depending on the induction of the expression of death receptor ligands or Bcl-2 family members, respectively [15].

Among post-translational modifications that can modulate FoxO proteins, phosphorylation is the best characterized [15]. FoxO phosphorylation has been reported to affect DNA binding activity and protein trafficking [44]. The available evidence supports that FoxO1 serine 256 phosphorylation—occurring in the DBD—impairs FoxO1 DNA-binding capability, thereby enhancing nuclear protein availability for additional phosphorylations [45]. Subsequent phosphorylation at the threonine 24 residue (together with serine 256 and the FoxO3 respective threonine 32 and serine 253) promotes FoxO binding by the 14-3-3 chaperone proteins, an event that—by modifying the conformation of FoxO nuclear localization signal—contributes to nucleus-to-cytoplasm shuttling [46]. Under some circumstances, using mutagenesis approaches FoxO1 threonine 24 phosphorylation was shown as necessary and sufficient for 14-3-3 binding [47]. Protein phosphatase 2A (PP2A) activity, targeting exactly threonine 32 and serine 253, has been shown to promote detachment between 14-3-3 and FoxO3 [48], and Dual Specificity Phosphatase 6 (DUSP6) potential in removing serine 256 FoxO1 phosphorylation has been evidenced [49].

### 3.1. FoxOs and Response to Conventional Antitumor Agents

In humans, FOXOs were originally discovered in rhabdomyosarcomas and acute myeloid leukemias, in which three members (FOXO1, FOXO3 and FOXO4) were identified due to the chromosomal translocations that produce fusion proteins [34]. The DNA translocations generating a fusion between the transcription domain of FOXOs and the DNA binding domain of other partners result in tumorigenesis via fusion protein-mediated gain of function and/or loss of FOXO tumor suppressor functions. These aberrant proteins can impact on transcriptional efficiency in turn deregulating tumor suppressive function with a possible effect on cellular response to drugs. In alveolar rhabdomyosarcoma (ARMS), the FOXO1 gene (13q14.11) is fused to either PAX3 (t(2;13)) or PAX7 (t(1;13)) genes [50], and the chromosomal translocation involving the MLL gene and FOXO3 (6q21) or FOXO4 (Xq13.1) genes (t(6;11); t(X;11)) have been found in secondary leukemia and acute lymphoblastic leukemia, respectively [51,52]. In those contexts, the fusion proteins appear to have a pathogenetic function. The pivotal role played by FoxO transcription factors is corroborated by the finding that in humans the FOXO1 gene is positioned within a DNA region that is deleted in prostate cancer, and reduced levels of FoxO1 are associated with prostate cancer as well as Ewing’s sarcoma [53,54]. In addition, in colorectal cancer, reduced levels of FoxO4 have been found to inversely correlate with miR499-5p up-regulation [55]. Moreover, deletion of FOXO1, FOXO3, and FOXO4 in mice results in a cancer-prone phenotype with development of thymic lymphomas and hemangiomas [56].

Although numerous studies have been published in the field, only a few report the response to chemotherapy in relation to the chromosomal translocations. Poor response to conventional chemotherapy and low survival rates are shown for patients suffering from ARMS and, despite the several treatment strategies considered, moderate improvements have been achieved. Various compounds such as the atypical retinoid ST1926, the histone deacetylase inhibitor entinostat, the proteasome inhibitor bortezomib, the Gsk3 inhibitor TWS119, as well as the topoisomerase I poison camptotecin were found to be able to reduce the level of the fusion protein Pax3-FoxO1 in ARMS [57,58,59,60,61]. Preclinical studies in ARMS treated with ST1926 have shown that the reduced expression of the oncoprotein correlated with early DNA damage, cell cycle arrest and apoptosis in turn resulting in growth inhibition [57]. Moreover, reduced levels of the fusion protein have been reported for ARMS cells treated with the histone deacetylase inhibitor entinostat. Of note, by direct transcriptional suppression of the Pax3-FoxO1 oncoprotein, entinostat demonstrated a remarkable caability to convert Pax3-FoxO1-positive into -negative ARMS cells [58]. Along this way, the authors of [59] identified the topoisomerase I poison camptothecin and the Gsk3 inhibitor TWS119 as ARMS-selective inhibitors, capable of inhibiting the oncoprotein function. Camptothecin, by significantly decreasing the level of Pax3-FoxO1, efficiently inhibited proliferation and induced apoptosis of ARMS cells [59]. A different mechanism of action has been proposed by the same research group for the Gsk3 inhibitor. This compound acts on Pax3-FoxO1 activity by indirectly regulating the phosphorylation status of the oncoprotein. The down-regulation of Gsk3 mediated by TWS119, reduced the phosphorylation, and in turn the transcriptional activity, of Pax3-FoxO1, with an inhibitory effect on cell growth and induction of apoptosis [60]. In addition, in ARMS cells treated with the proteasome inhibitor bortezomib, Marshall et al. [61] reported that the pro-apoptotic BH3-only family member Noxa is upregulated by the Pax3-FoxO1 oncoprotein and that the enhanced expression renders fusion protein-expressing cells more sensitive to apoptosis induced by bortezomib. Based on this finding the authors proposed the level of Noxa as a key determinant of ARMS biology able to predict tumor response to bortezomib [61].

#### 3.1.1. FoxO1 and Cellular Response to Conventional Chemotherapy

**Figure 2:**
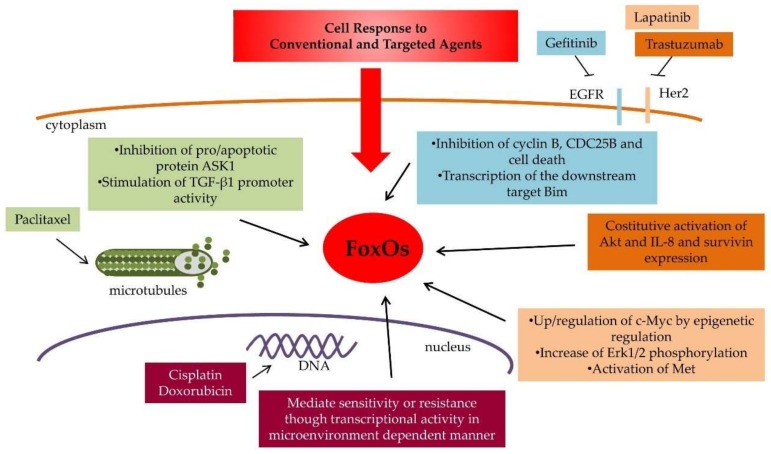
FoxO functions in cell response to antitumor drugs. The main cellular pathways regulated by FoxOs and involved in drug response and resistance of conventional and target-specific antitumor drugs are reported. The drug and the corresponding cellular response pathway are shown in different colors.

Several lines of evidence suggest that FOXO1 is implicated in response to conventional cytotoxic agents (Figure 2 and Table 1).

In ovarian carcinoma cells resistant to the microtubule stabilizer paclitaxel, increased levels of FOXO1 and the small redox protein thioredoxin (Trx1) have been described [62]. Specifically, a link between Trx1 and FOXO1 is observed in cells in which Trx1 bound to FoxO1 enhances FOXO1 transcriptional activity [62]. Of note, Trx1 plays a regulatory role via protein–protein interaction by binding to and inhibiting pro-apoptotic proteins, such as Apoptosis Signal-Regulating Kinase 1 (ASK1) [75]. Functional studies supported a role for FOXO1 in TRX1-induced resistance to paclitaxel which is lost in a TRX mutant (C69) poorly detectable in the nucleus [62]. The association between increased FOXO1 levels and paclitaxel resistance is in keeping with a recent report proposing a tumor suppressive role for Akt-phosphorylated FoxO1 in the cytoplasm [76]. In fact, FoxO1 nuclear localization, following exposure to taxane or PI3K inhibitors, was shown to increase the phosphorylation of the survival kinase Erk1/2 leading to drug resistance. When phosphorylated at serine 319 by Akt, FoxO1 binds to the scaffold protein IQGAP1, which integrates multiple signals acting as a critical regulator for MAPK activation [76]. Such a binding prevents Erk1/2 IQGAP1-dependent phosphorylation. In addition, low levels of FOXO1 in cell lines are associated with Erk1/2 increased activation. Of note, in this study, a FoxO1-derived phospho-mimicking peptide was shown to impede taxane-induced chemoresistance [76]. Thus, Akt-mediated phosphorylation of FoxO1 appears to be a mechanism to block FOXO1 tumor suppressor function in the nucleus and to activate a non-genomic tumor suppressor function of FoxO1 resulting in inhibition of the MAPK cell survival pathway.

In ovarian carcinoma preclinical models, FoxO1 contributes to the efficacy of the combination of cisplatin and selinexor, a selective inhibitor of the nuclear export receptor (XPO1)/Chromosome Region Maintenance-1 (CRM1) [63], because a stronger synergistic interaction was observed in cells expressing FoxO1 than in cells in which FoxO1 was down-regulated. XPO1 participates in the nuclear export of tumor suppressor and cell cycle regulating proteins, including FoxOs. Indeed, selinexor-induced enrichment of FoxO1 nuclear localization was exploited to increase cisplatin sensitivity in ovarian carcinoma cells [63]. Thus, inhibition of XPO1 favors FoxO1 nuclear localization and stimulates cisplatin-induced cell death. Importantly, the synergistic effect observed in vitro results into a potentiation of the antitumor activity in vivo [63].

In ovarian carcinoma cell models resistant to cisplatin, a down-regulation of FoxO1 levels has been reported following whole genome expression analysis [64]. Such a feature was associated with decreased expression of apoptosis-inducing genes. FOXO1 down-regulation was also linked to a decreased efficacy of the drug combination between cisplatin and a MEK (MAPK ERK Kinase) inhibitor, whereas FoxO1 appeared to be a determinant of the interaction between cisplatin and the inhibitor in parental cells [64]. In fact, dephosphorylation of serine 256 of FoxO1 was observed after MEK inhibition and exogenous expression of FoxO1 in resistant cells favors the synergistic interaction, thereby supporting a model where cell propensity to undergo apoptosis is stimulated by FoxO1 [64].

A critical role for Akt-mediated FoxO1 phosphorylation in regulation of resistance to chemotherapeutic agents is supported in hepatocellular carcinoma cells, in which the thyroid hormone (TH) bound to its receptor triggers survival signals [68]. This axis mediates resistance to chemotherapy (cisplatin and doxorubicin) by negative regulation of the pro-apoptotic Bim, whose expression is transactivated by FoxO1 [68]. Moreover, FoxO1 activity was found to be suppressed by the TH through transcriptional down-regulation and FoxO1 nuclear exclusion as a consequence of Akt-mediated phosphorylation, at an unknown residue [68].

In esophageal squamous cell carcinoma, the expression and activation of FoxO1 is influenced by the cross-talk between cancer-associated fibroblasts (CAFs) and tumor cells [67]. CAFs confer resistance to cisplatin and paclitaxel via secretion of Transforming Growth Factor-Beta 1 (TGFβ1) because inhibition of this process resulted in chemo-sensitization [67]. Besides, tumor cells produced TGFβ1 and promoted the activation of CAFs by up-regulating α-smooth muscle actin expression [67]. Of note, FoxO1 could stimulate TGFβ1 promoter activity resulting in increased TGFβ1 expression when FoxO1 was phosphorylated at threonine 24, thereby linking CAF-mediated resistance to a FoxO1-TGFβ1 loop [67]. Thus, FoxO1 participates in an autocrine/paracrine loop in preclinical models of drug resistance mimicking the tumor–microenvironment interplay.

The recent literature supports that FOXO1 is a target of several miRNAs, which may therefore take part into regulation of drug response, by affecting the levels of FOXOs mRNAs.

In a breast cancer cell model, including cells resistant to doxorubicin, miR-222 has been reported to confer drug resistance through FoxO1 [69]. The specific pathway leading to drug resistance appeared to implicate Phosphatase and Tensin Homolog (PTEN)/Akt [69]. Specifically, a negative correlation between miR-222 and FOXO1 expression was found upon transfection of miR-222 mimics, and inhibitors. which reduced and increased the levels of FOXO1 mRNA and protein, respectively [69]. The modulation of miR-222 levels alters FoxO1 localization, and increased nuclear FoxO1 levels are observed upon transfection with miRNA inhibitors [69]. Again, since the miR-222 up-regulation promoted Akt phosphorylation, partially decreasing cell sensitivity to doxorubicin, and pharmacological inhibition of Akt increased FoxO1 expression and drug sensitivity, miR-222-modulation of drug sensitivity likely depends on Akt/FoxO1 pathway activation [69].

A role for FoxO1 in mediating chemosensitivity of nasopharyngeal carcinoma has been demonstrated in a study reporting that miR-3188 regulates proliferation and chemosensitivity of this tumor type [73]. Indeed, an atypical miR-3188-mTOR–p-PI3K/AKT-c-JUN feedback loop modulated by FoxO1 was shown with direct targeting of mTOR by miR-3188, and inactivation of p-PI3K/p-AKT/c-JUN FoxO1—by suppressing such signaling—inhibited proliferation and sensitized cells to 5-fluorouracil, but not to cisplatin. The lack of an effect on cisplatin sensitivity was likely due to the concomitant activation of Zinc finger E-box binding protein 2 (ZEB2), a gene involved in the EMT process [73].

In contrast to a role for FoxO1 in favoring drug-induced apoptosis is the evidence of increased nuclear FoxO1 levels and DNA binding activity in breast cancer cells resistant to doxorubicin [70]. The mechanism of resistance of these cells, displaying a multi-drug resistant (MDR) phenotype, involves the over-expression of the ABCB1 gene, coding for P-glycoprotein. The ABCB1 gene promoter contains a putative binding site and transcription of the gene is stimulated by FOXO1 over-expression [70]. Functional studies with small interfering RNAs towards FOXO1 reversed doxorubicin resistance and decreased ABCB1 expression, further corroborating a role for FoxO1 activation in supporting the MDR phenotype [70].

#### 3.1.2. FoxO3 and Cellular Response to Conventional Chemotherapy

Another member of the FoxO protein family which has been recognized to play a critical role in drug response and resistance to conventional antitumor agents is the FOXO1 paralog gene FOXO3 (Forkhead Box O3, alias FOXO3a) (Figure 2 and Table 1). Similar to FOXO1, FOXO3 has been reported to regulate the transcription of the ABCB1 gene [71]. FOXO3 activation, which induces ABCB1 expression, enhances P-glycoprotein-mediated efflux in doxorubicin-resistant leukemic cells, thereby promoting cells survival [71]. In colon carcinoma cells, FOXO3 mediates the cytotoxic effect of cisplatin, through a mechanism involving FoxO3 dephosphorylation at threonine 32 and nuclear translocation, as well as induction of target genes, such as KIP and BIM [65]. These phenomena were evident in sensitive, but not in resistant cells. In fact, cisplatin-induced FoxO3 de-phosphorylation paralleled decreased Akt activation in sensitive cells [65].

In neuroblastoma cells, FoxO3 has been implicated in sensitization to apoptosis induced by the Top2 inhibitors doxorubicin and etoposide [72]. Inhibition of the PI3K/Akt pathway resulted in FoxO3 nuclear translocation associated with repression of the antiapoptotic survivin. Functional approaches supported the capability of FoxO3 to repress survivin transcription and expression and highlighted the relevance of survivin as regulator of cell death, because inhibition of multiple steps of FoxO3A-induced cell death was observed upon survivin over-expression, whereas FOXO3-induced apoptosis was accelerated upon survivin knockdown [72].

Consistently with a pro-apoptotic role for FoxO3, FoxO3 deficiency was shown to render colangiocarcinoma resistant to cisplatin in in vivo preclinical models [66]. The mechanism behind this finding involves Nuclear-related factor 2 (Nrf2), whose levels are regulated by the ubiquitin proteasome pathway. FoxO3, by controlling the transcription of Kelch-like ECH-associated protein 1 (Keap1) which acts as an adaptor of Cullin 3 (Cul3), the ubiquitin ligase complex that targets Nrf2, suppresses tumorigenesis and favors cisplatin-induced apoptosis [66].

A recent elegant study shows how different neuroblastoma cell lines respond to the activation of an ectopic FOXO3 allele [77]. Cell outcome following activation of the ectopic FoxO3 protein was different in various cell lines, with cells undergoing apoptosis or increasing their survival. The former cells (i.e., the apoptotic ones) carried mutant p53s, a condition resulting in abrogation of the p53-FoxO3 interaction. Conversely, in wild-type TP53 cells, the FoxO3-p53 dimers prevented the binding of FoxO3 to the promoter of the pro-apoptotic gene BIM [77]. Thus, the pro-survival role of FoxO3 in neuroblastoma appears to depend on target-gene regulation.

In addition, FoxO3 has been identified as a binding partner of Rab Escort Protein 1 (REP1), a cofactor of Rab geranyl-geranyl transferase 2, which regulates vesicles trafficking [74]. REP1, which has been implicated in maintaining cell survival in zebrafish tissues, was shown to block the nuclear translocation of FoxO3 through physical interaction leading to reduced FOXO3-induced apoptosis. Of note, over-expression of REP1 reduced 5-fluorouracil-induced apoptosis. REP1 negatively regulated the transcriptional activity of FOXO3 and the expression of FOXO3-target genes such as BAK, BIM and KIP. This study identifies REP1 as a regulator of nuclear-cytoplasmic shuttling of FoxO3 [74].

The role of FoxOs in drug response regulation is not limited to conventional antitumor agents. 

### 3.2. Role of FoxOs in Response and Resistance to Target Therapy.

Target therapy, which allows to hit specific factors implicated in promoting carcinogenesis, tumor growth and progression, can display reduced efficacy mainly because of the activation of compensatory or adaptive mechanisms that protect cancer cells. Among these mechanisms, the activation of FoxO-dependent pathways, including PI3K/Akt, plays a pivotal role. Therefore, FOXOs and related cellular pathways represent important determinants of response to target therapy (Figure 2 and Table 2).

In breast cancer cells, gefitinib has been reported to reduce Forkhead Box M1 (FoxM1) levels through the activation of FoxO3a, that in turn results in inhibition of cyclin B, CDC25B and cell death [78,87]. As a consequence of gefitinib-mediated dephosphorylation of Akt, a nuclear accumulation of FoxO3a with cell cycle arrest and apoptosis was observed in breast cancer cells exposed to the drug [78]. This feature was not evidenced in gefitinib-resistant breast cancer cells in which phosphorylated FoxO3a did not translocate into the nucleus, thereby negatively impacting on gefitinib antiproliferative activity [78]. The nuclear translocation of FoxO1 has been associated with the upregulation of c-Myc via the recruitment of epigenetic regulators, including the Myeloid/Lymphoid Leukemia 2 (KMT2D) and the histone acetyltransferase GCN5, that result in reduced sensitivity to the HER2 EGF-R dual kinase inhibitor lapatinib in Her2-overexpressing breast cancer cells [81]. More recently, an alternative mechanism played by FoxO1 and FoxO3 in lapatinib response of breast cancer cells has been reported [81]. Increased Erk1/2 phosphorylation induced by lapatinib is correlated with increased stability of c-Myc, known to be stabilized by Erk1/2-mediated phosphorylation [81]. The chronic inhibition of Her1/2 by lapatinib triggers a feedback loop activating the RAF/MEK/ERK pathway, in a FoxO-dependent manner. [88]. Again, several lines of evidence suggest a critical role for FoxO members in trastuzumab sensitivity in breast cancer cells. In fact, FoxO1 and FoxO3 down-regulation as well as constitutive activation of Akt result into increased expression of IL-8 and of the antiapoptotic protein survivin, both features that favor cell survival [83]. In addition, trastuzumab was reported to induce miRNA-542-3p expression, which mediates G1/S checkpoint arrest and in turn reduces proliferation in breast cancer cells [84]; conversely, the down-regulation of miRNA-542-3p has been associated with trastuzumab resistance via PI3K/Akt pathway-mediated activation of FoxO1a level [84]. The cellular response to the anti-estrogen tamoxifen was shown to be modulated by FoxO1-dependent transcriptional regulation of ABCC2, which codes for the multidrug resistance-associated protein 2 (MRP2) [85]. Indeed, tamoxifen-resistant breast cancer cells over-expressed MRP2 that in the promoter region contains four FOXO binding sites, a finding that renders FoxO1 a regulator of MRP2-mediated resistance [85]. In this context, nuclear localization of FoxO1 following deacetylation by Sirt1 is critical for stimulation of ABCC2 gene expression that impacts on tamoxifen resistance [85]. More recently, Vaziri-Gohar and coworkers [86] reported that FoxO1 acts as a positive regulator of Insulin-like Growth Factor Binding Protein 1 (IGFBP-1) transcription, which in turn inhibits IGF1-dependent signaling, critical for growth of normal breast cells and contributing to carcinogenesis, as well as to tamoxifen action. Consistently, decreased FoxO1 levels were observed in breast cancer tamoxifen-resistant cells [86].

In lung cancer, the authors of [79] reported a correlation between high levels of FoxO3a and gefitinib response in patients [79]. Moreover, a JNK-mediated up-regulation of FoxO1, FoxO3 and FoxO4 regulating the transcription of the downstream target BIM, critical for apoptosis induction by the EGFR inhibitor AG1478, has been reported in PC-9 lung cancer cells [80].

FOXO1 downregulation has been reported to play a role also in Her2-positive gastric cancer cells resistant to lapatinib [82]. In these cells, the acquisition of lapatinib resistance seems to depend on the activation and up-regulation of the RTK Met via FoxO1 decrease [82].

## 4. Conclusions

Several studies have provided evidence of an involvement of FoxOs in cellular processes that influence cell response to antitumor agents. In cancer cells, FoxOs have been shown to inhibit cell proliferation and to activate the expression of death ligands and apoptotic genes as well as of genes involved in DNA repair [4]. Of note, survival kinases (e.g., Erk1/2 and Akt) phosphorylate FoxOs and inhibit the protein activity [89,90]. However, the significance of FoxOs in tumor cell fate has not been completely elucidated and cannot be simplified. Indeed, the basal levels of FoxOs in resistant cells do not exhibit consistent patterns of modulation among different studies and both down- and up-regulation has been reported in resistant cells [71,91]. The link between increased expression of FoxOs and drug resistance may be dependent on FoxO engagement in the PI3K/Akt pathway. In this regard, the continuous triggering of FOXOs by cytotoxic drugs, such as doxorubicin, increases survival through a feedback loop involving the induction of PIK3CA and hyperactivation of the PI3K/Akt pathway [71]. An impairment of the ability of cisplatin to activate FoxO signaling has been reported both for FoxO1 in ovarian carcinoma cells [64] and FoxO3 in colon cancer cells [65]. However, this phenomenon can be regarded as a sort of vulnerability exploited to restore FoxO signaling with inhibitors of nuclear export, as shown for FoxO1 with the XPO1 inhibitor selinexor in combination with cisplatin in ovarian cancer cells or for FOXO3 with psammaplysene in colon cancer cells [64,65]. Several mechanistic details regarding FoxO1 action, for instance, in suppressing tumor cells growth have not been clarified yet, but under most circumstances they seem to involve the Akt pathway which appears critical in clinical specimens. In fact, activated Akt correlates with phospho-FoxO1 and with phosphorylation of EGF receptor in nasopharyngeal carcinoma [92]. It is likely that the complex regulation of the FOXOs function rely also on the pattern of phosphorylation of these proteins that has been only in part unraveled. In fact, although it is clear that FoxOs are mainly phosphorylated by Akt, JNK and AMPK, other kinases (e.g., ATM, Erk1/2, etc.) have also been reported to phosphorylate FoxOs [5], likely making cell response to drug dependent on the expression of such kinases in different tissues. A further difficulty in the understanding of the precise role of FoxOs in tumor cell response may be due to the redundant function of FOXOs, a behavior supported by the phenotypes of knock-out mice in terms of tumor suppressor function. Indeed, knocking out a single FOXO gene does not result in increased tumor incidence and two FOXO knock-outs have to be combined to observe increase tumor incidence in aged mice [5]. Functional redundancy may in principle also affect cell response, when considering the heterogeneous nature of tumors.

In addition, to explain the paradox governing the regulation of stress resistance or FoxO-mediated pro-apoptotic gene expression, a threshold of stimulus intensity has been proposed as a switch allowing distinction between the two opposite functions [93]. Depending on the intensity of the stimulus, FoxO activates the expression of stress-resistance genes (below the threshold) or pro-apoptotic factors (over the threshold) [93] that guide cell fate. Moreover, the cell type is crucial for FoxO functions since different gene expression patterns, typical of cell histology, seem to orchestrate apoptosis in some cells (e.g., lymphocytes and neurons) or survival in others [93].

In conclusion, the tumor suppressor activity of FOXOs has been reported in a plethora of studies. The involvement of FoxOs in cell cycle arrest, cell death, senescence, invasiveness and metastasis, angiogenesis and oxidative stress response render the transcription factors intriguing to better understand the heterogeneous response of cancer cells to treatment, and under some circumstances, to design novel therapeutic approaches [64,76]. FoxO levels per se do not seem to explain drug resistance/sensitivity, but FoxO partners in specific molecular backgrounds appear to be critical to determine cell fate. Because FoxOs undergo multiple post-translational modifications, additional efforts are required to better understand their combined effects on the transcription factor activities.

## Figures and Tables

**Table 1:** FoxO role in cell sensitivity and resistance to conventional agents.

Tumor	Drug	Mechanism of Response		Ref
Ovarian carcinoma	paclitaxel	Increased levels and transcriptional activity of FoxO1.	Resistance	[62]
Ovarian carcinoma	cisplatin combined with selinexor	Enrichment of FoxO1 nuclear localization.	Sensitivity	[63]
Ovarian carcinoma	cisplatin	Down-regulation of FoxO1 levels.	Resistance	[64]
Colon carcinoma	cisplatin	FoxO3 dephosphorylation (threonine 32) and nuclear translocation.	Sensitivity	[65]
Colangiocarcinoma	cisplatin	Increased transcriptional activity of FoxO3 (up-regulation of Keap1) and activation of proteasome pathway.	Sensitivity	[66]
Esophageal squamous cell carcinoma	cisplatin, paclitaxel	FoxO1 stimulation of TGF-β1 expression.	Resistance	[67]
Hepatocellular carcinoma	cisplatin, doxorubicin	Suppression of FoxO1 activity (down-regulation of the pro-apoptotic Bim).	Resistance	[68]
Breast cancer	doxorubicin	FoxO1 reduced levels and nuclear localization.	Resistance	[69]
Breast cancer	doxorubicin	FoxO1 over-expression (up-regulation of ABCB1).	Resistance	[70]
Leukemic cells	doxorubicin	FoxO3 activation (stimulation of ABCB1 expression).	Resistance	[71]
Neuroblastoma	doxorubicin and etoposide	FoxO3-mediated reduction of survivin expression.	Sensitivity	[72]
Nasopharyngeal carcinoma	5-fluorouracil	miR-3188-mTOR-p-PI3K/AKT-c-JUN feedback loop modulated by FoxO1 sensitize cells.	Sensitivity	[73]
Colon carcinoma	5-fluorouracil	FoxO3 nuclear translocation (down-regulation BAK, BIM, KIP).	Resistance	[74]

**Table 2:** FoxO role in cell response to target specific agents.

Tumor	Drug	Mechanism of response		Ref
Breast cancer	gefitinib	FoxM1 reduction and FoxO3a nuclear accumulation	Sensitivity	[78]
Lung cancer	gefitinib	Increased FoxO3a level	Sensitivity	[79]
Lung cancer	AG1478	Up-regulation of FoxO1, FoxO3 and FoxO4	Sensitivity	[80]
Breast cancer	lapatinib	Nuclear translocation of FoxO1	Resistance	[81]
Gastric cancer	lapatinib	FoxO1-mediate upregulation of Met	Resistance	[82]
Breast cancer	trastuzumab	FoxO1 and FoxO3 down-regulation	Resistance	[83]
Breast cancer	trastuzumab	Increased FoxO1a level	Resistance	[84]
Breast cancer	tamoxifen	Nuclear localization of FoxO1 (ABCC2)	Resistance	[85]
Breast cancer	tamoxifen	Decreased FoxO1 levels	Resistance	[86]
